# Is There (Still) a Place for Sequential Conditioning?

**DOI:** 10.3390/curroncol32040196

**Published:** 2025-03-27

**Authors:** Boris Bours, Stavroula Masouridi-Levrat

**Affiliations:** Division of Hematology, Department of Oncology, Geneva University Hospitals, 1205 Geneva, Switzerland; boris.bours@hug.ch

**Keywords:** sequential, conditioning, sequential conditioning, allogeneic stem cell transplantation, acute myeloid leukemia, myelodysplastic neoplasia

## Abstract

There is still an unmet need for the treatment of high-risk hematological malignancies. To date, allogeneic stem cell transplantation remains the only chance of cure. Most patients suffering from high-risk hematological malignancies are of an older age and often present with comorbidities. Moreover, patients achieving remission often suffer from early relapse. Amongst the different treatment options, sequential conditioning has yet to prove its value against other conditioning regimens. Sequential conditioning relies on a short course of intensive chemotherapy that is quickly followed by immunosuppressive conditioning before allogeneic stem cell transplantation. Here, we will try to determine which patients can benefit from sequential conditioning. Amongst the different sequential regimens, we will also try to assess if one regimen is better than all the others. Despite the several studies conducted on sequential conditioning, very few are prospective work and head-to-head comparisons are almost inexistant. Sequential conditioning also relies on the use of prophylactic donor lymphocyte infusion post-transplantation. Hence, limiting non-relapse complications is of primary importance to the allow administration of post-transplant treatment. In the era of new targeting therapies, is there still a place for sequential conditioning? Can patients benefit from an association of new therapeutic agents and sequential conditioning?

## 1. Introduction

The therapeutic arsenal for acute myeloid leukemia (AML) is expanding and, currently, standard treatment with chemotherapy and other molecules can achieve complete remission (CR) as high as 89% [1].

However, primary refractory AML, defined by failure to achieve hematologic CR after one or two courses of induction chemotherapy [2,3], has a dismal prognosis. For those patients, allogeneic hematopoietic stem cell transplantation (allo-HSCT) remains the only curative option.

How to handle refractory disease before allo-HSCT is still unclear: should we delay transplantation, trying to achieve better remission with another round of intensive chemotherapy without any certainty of response, or should we carry on with transplantation in a prompt manner? In the latter case, which conditioning regimen should be used?

Regimens used in this setting consist mainly of myeloablative conditioning (MAC) protocols with disappointing results and significant toxicities.

In 2010, Duval et al. [4] analyzed the outcomes of 2255 patients with acute lymphoblastic or myeloid leukemia in relapse or in primary induction failure who received a first allo-HSCT between 1995 and 2004 after an MAC regimen. For the 1673 AML patients, mortality rate at day + 100 was 39%, while 3-year overall survival (OS) was only 19%, with leukemia being the main cause of death (42%). A retrospective analysis of the European Group for Blood and Marrow Transplantation (EBMT) compared two classic MAC regimens, busulfan i.v. plus cyclophosphamide (BuCy) vs. cyclophosphamide plus total body irradiation (CyTBI), for 852 patients with refractory AML, between 2000 and 2012 [5]. Outcomes were similar between the groups, with a 2-year OS of 31.2% with BuCy vs. 33.4% with CyTBI; the 2-year leukemia-free survival (LFS) rates were 25% and 28.4%, respectively, while CyTBI was associated with a lower non-relapse mortality (NRM) risk. Both studies showed better outcomes for patients treated for primary refractory AML than for those with active disease after first or second relapse [4,5].

The reduced-intensity conditioning (RIC) regimen, used for older and unfit patients, relies more on the graft-versus-leukemia (GvL) effect than on the myeloablative effect of high-dose chemotherapy. Though better tolerated, RIC is associated with a higher risk of relapse and an inferior OS [6]. Thus, in the case of active disease, it would not be sufficient to control the disease while waiting for the GvL effect to take over [7,8]. A recent Japanese study analyzed the outcomes of 707 patients (median age of 61 years, range 55–65) with relapsed/refractory (R/R) AML and who underwent allo-HSCT after an RIC regimen (fludarabine-melphalan/busulfan) [9]. The results showed a 2-year and 5-year OS of 30.1% and 22%, respectively, with the majority of patients experiencing a relapse in the first two years. The cumulative incidence of relapse (CIR) was 52.5% at 2 years and 53.6% at 5 years, while the 2-year and 5-year NRM rates were 22.6% and 27.5%, respectively, resulting in a poor 5-year Graft-versus-Host Disease (GvHD)-free, relapse-free survival (GRFS) of 13.6%. In Table 1, we show the outcomes for patients with active disease treated with different conditioning regimens.

As shown above, allo-HSCT for active disease has poor outcomes after both RIC and MAC regimens. The level of disease response before allo-HSCT is of great importance: a high count of circulating blasts or bone marrow blasts > 20% are both associated with decreased OS and LFS and a high treatment-related mortality [12,13]. Patients with refractory disease and a blast count > 5% have a significantly worse outcome in terms of OS and LFS than that of patients with CR but with positive measurable residual disease (MRD) [14,15]. Finally, pretransplant MRD-positivity is associated with higher risk of relapse than MRD-negativity [16,17]. For this reason, strategies to reduce the disease burden before transplantation have long been investigated.

## 2. Introduction of Sequential Conditioning

One of the options to reduce leukemic burden before allo-HSCT is sequential conditioning (SqC). The concept of SqC was introduced in 2005 by Schmid and Kolb [18]. Intensive chemotherapy is administered using the FLAMSA regimen (fludarabine 30 mg/m^2^, high-dose cytarabine 2 g/m^2^, and amsacrine 100 mg/m^2^ from days −12 to −9), followed by a three-day rest, and then RIC, consisting of 4 Gy TBI on day −5, cyclophosphamide (40 mg/kg for HLA-identical siblings, 60 mg/kg for unrelated or mismatched donors) on days −4 and −3, and rabbit antithymocyte globulin (ATG) (10 mg/kg for HLA-identical siblings, 20 m/kg for unrelated or mismatched donors) from days −4 to day −2. After transplantation, and if there are no contraindications, treatment is carried on with prophylactic donor lymphocyte infusion (pDLI).

The FLAMSA-RIC sequential conditioning process can be divided into several parts: an induction antileukemic chemotherapy, an immunosuppressive conditioning, and pDLI for immune restitution [19]. Amsacrine is used instead of anthracyclines, not only because it is less toxic, but also to avoid cross-resistance with anthracyclines as it belongs to another class of DNA-intercalating drugs and inhibitors of topoisomerase II [20]. The use of the first sequential conditioning showed promising results for this high-risk cohort, with an 88% CR rate at 30 days after transplant, a 2-year LFS of 40%, and a 2-year OS of 42% [18]. In Table 2, we display the outcomes after different types of sequential conditioning regimens.

To our knowledge, the FIGARO trial is the only prospective trial comparing SqC to standard RIC [21]. Two hundred and forty-four patients with AML or high-risk myelodysplastic neoplasm (MDS) received either sequential conditioning (FLAMSA-Bu) or RIC (Flu-Bu-ATG, Flu-Mel-alemtuzumab, or Flu-Bu-alemtuzumab). Ninety-four percent of AML patients were in CR1 or CR2 and only 5% were in a primary refractory state. An interesting part of the analysis is the integration of multiparameter flow cytometry (MFC) to define MRD in the pre-transplant setting and at day 42 post-transplant. The 2-year OS was similar in both arms, 58.8% in the control arm, and 60.9% in the FLAMSA-Bu arm (hazard ratio (HR) 1.05; 85% CI; 0.80–1.38; *p* = 0.81), and so was the 2-year EFS: 48.7% and 54.2%, respectively (HR 0.96; 95% CI; 0.68–1.35; *p* = 0.82). The 1-year treatment-related mortality (TRM) was 16.8% in the RIC arm and 20.5% in the SqC arm (without significant statistical difference) and no statistically significant difference was observed in terms of acute-GvHD (aGvHD), chronic-GvHD (cGvHD), and CIR. Pre-transplant MRD-positivity was associated with higher risk of relapse and reduced OS but no difference was observed in post-transplant MRD clearance at day + 42 between both groups. The acquisition of full donor T-cell chimerism was also similar in control and experimental arms. In our opinion, even though FIGARO trial has not shown the superiority of SqC, regardless of pretransplant MRD status, no conclusion can be made regarding patients with active disease, as only nine patients were in a primary refractory state. Therefore, robust data comparing RIC and SqC in patients with active disease at the time of transplantation are still lacking.

Very few other studies have compared FLAMSA-RIC to RIC. An EBMT analysis [22] compared a conditioning regimen with fludarabine-treosulfan (Flu-Treo) (RIC or MAC) to FLAMSA-Busulfan and FLAMSA-TBI in patients with AML in their first or second remission. The FLAMSA-based groups showed a better LFS but an increased risk of aGvHD. However, similar rates of OS and NRM were observed. Between the two FLAMSA groups, FLAMSA-TBI was better than FLAMSA-Busulfan in terms of CIR, LFS, OS, NRM, and aGvHD incidence. This corroborates the safety and efficacy of FLAMSA-RIC conditioning, even though, once again, the patients were in disease remission. A single-center German analysis [23] of 113 consecutive patients retrospectively compared the Flu-Treo regimen (treosulfan at 42 g/m^2^ for patients < 60 years and 36 g/m^2^ for patients > 60 years) to FLAMSA-RIC (modified RIC part with busulfan instead of TBI). Given an uneven distribution concerning remission status at transplant (51% of the patients in the Flu-Treo group being in CR1 or 2 vs. 22% in the FLAMSA-RIC group), we can conclude that Sqc is at least non-inferior as no differences were observed in 2-year OS (Flu/Treo 45% and FLAMSA-RIC 41%), LFS (Flu-Treo 11 months and FLAMSA-RIC 10.5 months), TRM (Flu-Treo 32% and FLAMSA-RIC 29%), and GvHD incidence between the two groups.

### “Next-Generation” Sequential Conditioning Regimens

Despite the absence of prospective studies comparing SqC to RIC in patients with active disease, the FLAMSA-RIC regimen is still widely used in the context of R/R AML. Since the introduction of FLAMSA-RIC twenty years ago, several studies have shown the feasibility and the relative safety of SqC, and other protocols have been proposed to minimize toxicities while still achieving a significant anti-leukemic effect.

For example, in a prospective phase 2 study [24] (Table 2) including 24 patients with primary refractory AML, a clofarabine-cytosine arabinoside debulking regimen was used, followed by RIC comprising busulfan, cyclophosphamide, and ATG, while pDLIs were administered post-transplant. Clofarabine replaced amsacrine and busulfan was preferred to TBI to diminish toxicity. With a 2-year NRM of 12%, the limitation of treatment-related toxicity was successful, while still retaining disease control (2-year OS 38% and 2-year LFS 29%). In 2020, however, a French retrospective study of 131 patients could not achieve the same results using the same sequential regimen [25]. The worse outcome can be explained by an older and more heavily pretreated population, with more patients being included with active disease relapse instead of those in a primary refractory state.

The TEC-RIC (thiotepa, etoposide, cyclophosphamide) regimen is another SqC regimen which has been evaluated for patients with R/R hematological malignancies. A retrospective study compared the outcomes for 72 patients treated with TEC-RIC between 2013 and 2016 [26] (Table 2). Most patients had R/R AML (n = 44) or R/R ALL (n = 7), and most of them had active disease. The population was heavily pretreated; the Karnofsky performance status was < 90% for 54% of the patients. However, outcomes were favorable when compared to other SqC regimens. The use of this broad-spectrum chemotherapy as “debulking” chemotherapy in association with RIC (fludarabine, i.v. busulfan, and ATG) and post-transplant cyclophosphamide (PTCy) showed promising results in the haploidentical group. This sub-population had a 2-year relapse incidence of 35.9%. The use of ATG in the haploidentical group resulted in a low incidence of acute and chronic GvHD without compromising the outcomes for R/R patients.

**Table 2:** Sequential conditioning studies.

Authors	Patients	Cytoreduction—Conditioning	Underlying Disease	Disease Status	CR at D + 30	OS	NRM	LFS/DFS—EFS—RFS—GRFS	Relapse	GvHD	Patients Receiving pDLI (%)
Schmid et al. (2005) [18]	N = 75 Median age: 52.3 (range: (18.5–65.8))	Original FLAMSA + RIC	AML, MDS	Refractory AML (n = 27); Untreated AML relapse (n = 22); Progressive MDS (n = 10); Remission (n = 16).	88%	42% at 2 years	33% at 1 year	LFS at 2 years: 40%	n = 15 at a median of 149 days (range, 65–770 days)	aGvHD: n = 46 (61%) (G III n = 12, Grade IV n = 6) cGvHD: n = 26 (45%) (extensive n = 11)	n = 12
Mohty et al. (2017) [24]	N = 24 Median age: 47 (range: 20–57)	Clofarabine, cytarabine + RIC (Bu/Cy, ATG)	AML	Primary refractory	75%	38% at 2 years	12% at 2 years	LFS at 2 years: 29%	54.2% at 2 years	aGvHD: n = 4 (G III n = 1, Grade IV n = 0) cGvHD: n = 9 (extensive n = 4)	n = 6 (25)
Bourgeois et al. (2020) [25]	N = 131 Median age: 52.6 (range: 18–71)	Clofarabine, cytarabine + RIC (Bu/Cy, ATG)	AML, MDS, MPN	Primary refractory (n = 81); First or second relapse (n = 46); Unknown (n = 4).	63%	22% at 2 years	35.1% at 2 years	DFS at 2 years: 19.5%; GRFS at 2 years: 14%.	45.4% at 2 years	Grade II–IV aGvHD: 29.7% Grade III–IV aGvHD: 10.4% cGvHD: 21.4% Moderate/severe cGvHD: 6.2%	NM
Zoellner et al. (2015) [27]	N = 16 Median age: 53 (range: 23–66)	Clofarabine + RIC (Flu/Cy/Mel) + PTCy	B- or T-cell Non-Hodgkin’s lymphoma	Disease progression (69%); Stable disease (6%); Partial response (25%); CR (0%).	CR: n = 4 PR: n = 11	68.8% at 2 years	18.75% at 1 year	PFS at 2 years: 50%	Unclear	aGvHD: n = 10 (G I n = 4, G II n = 5, G III n = 1) cGvHD: n = 4 (mild)	0
Duléry et al. (2018) [26]	N = 72 Median age: 54 years (range: 16.5–72)	Thiotepa, etoposide, cyclophosphamide + RIC (Flu/Bu, ATG) ± PTCy	AML, ALL, MDS, MPN, CMML, lymphoma	Refractory disease (>6% marrow blasts in 34/51 patients with acute leukemia (AML or ALL)).	95.7%	46.4% at 2 years *AML patients:* 41.5% at 2 years	23.7% at 2 years *AML patients:* 15.9% at 2 years	EFS at 2 years: 38.9% GRFS at 2 years: 28.7% *AML patients:* EFS at 2 years 37.5%; GRFS at 2 years: 21.9%.	At 2 years: Haplo: 35.9%; MRD: 31.2%; UD: 43.1%.	Grade II–IV aGvHD: 23.6% Grade III–IV aGvHD: 13.9% Haplo: 3.7%; MRD: 0%; UD: 31% cGvHD: 32.1%	n = 13 (18)
Tischer et al. (2013) [28]	N = 18 Median age: 39 (range: 20–69)	Clofarabine + RIC (Flu/Cy) + Mel or Treo or Eto or TBI + PTCy	AML or ALL	Active disease: Relapse (78%); Refractory (22%).	77.8%	55.5% at 1 year	23% at 1 year	RFS at 1 year: 39% (95% CI, 18–60)	44.4% at a median time of 5.6 months	aGvHD Grade II– IV: 50%; Grade III– IV: 11%. cGvHD: 27.7% (non-severe)	NM
Fraccaroli et al. (2018) [29]	N = 33 Median age: 58 (range: 32–71)	FLAMSA + RIC (Flu/Cy +Mel or TBI4) + PTCy Clofarabine + RIC (Flu/Cy + Mel or TBI4) + PTCy	High-risk and R/R AML, high-risk MDS	Active disease: Relapsed (55%); Refractory (21%); Untreated (24%).	97%	1 year: 56%; 2 years: 48%; 3 years: 48%.	1 year: 15%; 3 years: 15%.	DFS: 1 year: 49%; 2 years: 46%; 3 years: 40%.	1 year: 28% 2 years: 35% 3 years: 35%	aGvHD: Grade II–IV 24%; Grade III–IV 3% cGvHD: 38%; CI of severe cGvHD 3.7%.	0
Doppelhammer et al. (2019) [30]	N = 68 Median age: 54 (range: 28–71)	HLA-matched (n = 34) FLAMSA + RIC (unspecified with ATG) Haplo (n = 34) Clofarabine or FLAMSA + RIC (unspecified) + PTCy	High-risk AML	PIF (18%); Relapse (60%); Untreated (9%); CR (13%).	91% for HLA-matched 94% for Haplo	56% at 3 years	16% at 3 years	LFS at 3 years: 49% GRFS at 3 years: 34% for Haplo; 28% for HLA-matched. cGRFS at 3 years: 40% for Haplo; 37% for HLA-matched.	34% at 3 years	aGvHD, Grade ≥ II: 50% after HLA-matched; 13% after Haplo cGvHD; 35% after HLA-matched; 27% after Haplo. Moderate/severe cGvHD: 24% after HLA-matched; 15% after Haplo.	NM
Jondreville et al. (2021) [31]	N = 36 Median age: 55 (range: 26–69)	FLAMSA + RIC (Bu/Mel + ATG if PBSC) + PTCY for Haplo	AML (n = 29), MDS (n = 6), myelofibrosis (n = 1)	AML (n = 29): PIF (n = 13), active relapse (n = 10), CR with poor genetic/molecular markers (n = 5), untreated (n = 1). High-risk MDS (n = 6): Refractory (n = 4), relapse (n = 1), untreated (n = 1). Myelofibrosis (n = 1): Accelerated phase with PIF (n = 1).	/	62% at 2 years 2-year OS for HLA-matched: 89% 2-year OS for Haplo: 34%	HLA-matched: 0% at 2 years Haplo: 58% at 2 years	DFS at 2 years: 52% (entire cohort): 68% (HLA-matched); 34% (Haplo). DFS at 2 years: 37% (HLA-matched); 22% (Haplo).	HLA-matched: 32% Haplo: 20%	Grade III–IV aGvHD 56% (MUD) 53% (Haplo) 0% (MRD) Extensive cGvHD 25% (entire cohort) 22% (MUD) 18% (Haplo) 40% (MRD)	n = 7 (19)
Wang et al. (2018) [32]	N = 47 Median age: 42 (range: 16–62)	FLAG-Ida + RIC (Bu/Flu) + PTCY for Haplo or MUD	AML	PIF (n = 23) Early relapse (n = 1) R/R (n = 23)	89%	43.8 ± 7.8% at 3 years (estimated)	25.7 ± 4.2% at 3 years (estimated)	42.3 ± 7.8% at 3 years (estimated)	33.5 ± 5.7% at 3 years (estimated)	aGvHD: Grade II–IV: n = 15; Grade III–IV: n = 5. cGvHD: n = 15 Extensive: n = 10.	n = 13

Abbreviations: AML, acute myeloid leukemia; ALL, acute lymphoblastic leukemia; MDS, myelodysplastic neoplasm; MPN, myeloproliferative neoplasm; CMML, chronic myelomonocytic leukemia; CR, complete remission; LFS, leukemia-free survival; DFS, disease-free survival; EFS, event-free survival; RFS, relapse-free survival; GRFS, graft-versus-host disease-free, relapse-free survival; aGvHD, acute graft-versus-host disease; cGvHD, chronic graft-versus-host disease; pDLI, prophylactic donor lymphocyte infusion; RIC, reduced-intensity conditioning; Bu, Busulfan; Cy, cyclophosphamide; ATG, anti-thymocyte globuline; Haplo, haploidentical stem cell transplant; SCT, stem cell transplant; NM, not mentioned; Flu, fludarabine; PTCy, post-transplant cyclophosphamide; MRD, matched related donor; MUD, matched unrelated donor; Mel, melphalan; PR, partial remission; Treo, treosulfan; Eto, etoposide; TBI, total body irradiation; cGRFS, chronic graft-versus-host disease-free, relapse-free survival; PIF, primary induction failure.

In 2020, the Acute Leukemia Working Party of EBMT conducted an analysis comparing the outcomes of 2132 patients diagnosed with R/R AML in active disease (≥5% blasts in bone marrow) and treated with six different types of sequential conditioning [33]. They compared FLAMSA-TBI4 (FLAMSA-TBI4/Cy or FLAMSA-TBI4), FLAMSA-chemo (FLAMSA-Mel, FLAMSA-Treo/Cy, FLAMSA-Bu/Flu, FLAMSA-Bu/Cy), Mel-Flu/TBI8, Mel-Treo/Flu, Clo/ARAC-(Bu_2_/TBI4)/Cy, and Thio/ETO-Cy/Bu_2_/Flu. The latter was excluded from the following analysis due to the small population of the cohort. In the multivariate analysis, the main difference was a better outcome in terms of 2-year LFS for patients < 55 years treated with Mel-Flu/TBI8 (38.8% vs. 30.3% for the entire cohort). For patients > 55 years, 2-year LFS was similar for FLAMSA-chemo, FLAMSA-TBI4, and Mel-Flu/TBI8, and significantly better than that achieved by Mel-Treo/Flu (HR: 1.5; 95% CI; 1.1–2.1; *p* = 0.2) and Clo/ARAC-(Bu2/TBI4)-Cy (HR: 1.7; 95% CI; 1.2–2.4; *p* < 0.1). FLAMSA-based regimens were also associated with a significantly lower rate of NRM.

Overall, SqC conditioning is well tolerated, with encouraging results in terms of OS compared to MAC and RIC for patients with active disease [4,5,9,10,11] (Table 1). However, based on the available data, SqC does not show better outcomes for patients with AML in CR or for patients with positive MRD [21,22]. FLAMSA-based regimens and TEC-RIC seem to be better tolerated, with lower NRM compared to other SqC regimens. This seems to be particularly the case for FLAMSA regimens using a chemotherapy-based RIC in the older population [33,34]. We believe that toxicity can be reduced by using FLAMSA in combination with RIC using treosulfan. We also believe that reducing conditioning regimen toxicity and GvHD risk should be our priority, as it will allow the use of post-transplant therapies such as pDLI.

## 3. Acute Myeloid Leukemia with Active Disease

Focusing on the patients with AML with active disease, the EBMT Acute Leukemia Working Party performed a retrospective analysis of 267 patients with R/R AML undergoing first allo-HSCT after sequential conditioning [35]. Amongst those patients, 125 were in primary induction failure, 120 in first relapse, and 22 were in second relapse. The conditioning regimen used was FLAMSA, followed by RIC consisting of either TBI/Cy, Bu/Cy, busulfan alone, or melphalan alone. The 3-year OS for the entire cohort was 30.4%, but it drastically decreased to 13.6% for patients in their second relapse, emphasizing the idea that allo-HSCT should be performed promptly in the disease course. The 3-year NRM was 25.9%, with lower NRM for patients < 50 years of age and for patients receiving in vivo T-cell depletion (anti-thymocyte globulin or alemtuzumab). The 3-year probability of relapse was 48.5%, 3-year GRFS was 17.8%, and the 3-year LFS was 25.6%.

Those results, whilst poor, are superior to historical results for R/R AML treated with chemotherapy alone [4,36]. Recently, the use of venetoclax as an adjunct to FLAG-Ida chemotherapy has improved R/R AML outcomes, with a CRc rate of 67%, a median OS of 13 months [37], and 69% of patients achieving CR with negative MRD. Moreover, this treatment allowed transition to allo-HSCT for 46% of patients. R/R AML patients achieving CRc with negative MRD after FLAG-Ida+ venetoclax and undergoing allo-HSCT had an impressive 1-year survival post HSCT of 78%. With such good results for R/R AML patients, one may wonder if there is still a place for SqC. However, to our knowledge, there is no trial which compares the different strategies for R/R AML. If the achievement of remission is of paramount importance and FLAG-Ida-venetoclax is currently the best salvage treatment, the addition of another course of chemotherapy does not guarantee the achievement of remission and may lead to a loss of precious time and a needless risk of complications for patients with chemo-refractory AML. In the event of AML relapse, we may consider adding venetoclax to the SqC regimen, as prompt transplantation with SqC may offer a better chance than salvage chemotherapy before proceeding with allo-HSCT.

Encouraging results were also provided by a 2018 study that analyzed the outcomes of 47 patients with refractory AML [32]. The SqC regimen used was FLAG-Ida for the myeloablative part and busulfan/fludarabine for the RIC part. With a median follow-up of 24.3 months, the estimated 3-year OS was 43.8 ± 7.8% and the estimated 3-year NRM was 25.7 ± 4.2%.

Rodríguez-Arbolí et al. (2020) [38], in another retrospective registry-based analysis of EBMT ALWP, compared FLAMSA-RIC to MAC for younger patients with active R/R AML. A total of 1018 patients (median age 39 years, range 18–50) were included, of whom 258 received Bu/Cy, 314 received Cy/TBI, 318 received FLAMSA-TBI/Cy, and 128 received FLAMSA-CT (RIC regimen without TBI: Bu/Cy, melphalan, or busulfan alone). CR after allo-HSCT was obtained in 69% of patients after MAC, 76% after FLAMSA-TBI, and in 76% after FLAMSA-CT. FLAMSA-CT was associated with a significantly better 2-year OS of 50% (vs. 34% for CyTBI, 33% for BuCy, and 36% for FLAMSA-TBI; *p* = 0.03) and 2-year LFS (HR for FLAMSA-CT 0.73; 95% CI; 0.54–1.00; *p* = 0.046). Unsurprisingly, the 2-year LFS was lower for patients in their second relapse compared to those in their first relapse and with primary refractory disease (16% vs. 29% and 32% respectively). The 2-year NRM was significantly lower for the FLAMSA-CT group (7% vs. 18% for FLAMSA-TBI, 19% for TBI/Cy, and 16% for Bu/Cy). No significant differences were observed between the different conditioning regimens for engraftment, aGvHD, cGvHD, and GRFS.

However, a study by Société Francophone de Greffe de Moelle et de Thérapie Cellulaire (SFGM-TC) [39] did not find such a difference when comparing MAC to SqC (FLAMSA or clofarabine-cytarabine for the myeloablative part followed by RIC with TBI or with busulfan). Though aGvHD and cGvHD occurred at a significantly higher rate in the MAC groups, the analysis did not show any difference between MAC and SqC in terms of OS, LFS, CIR, and NRM.

Finaly, the EMBT ALWP also compared FLAMSA-RIC to two reduced-toxicity MAC regimens, Flu/Treo (FT) and thiotepa/busulfan_2-3-4_/fludarabine (TBF), in 856 patients with R/R AML [40]. Between FT, TBF, and FLAMSA-based regimens, no differences were observed concerning the 2-year OS (37%, 24%, 34%, respectively; *p* = 0.1). The incidence of GvHD, NRM, and CIR were also similar. Once again, ATG use was associated with a significantly lower risk of acute and chronic GvHD and a tendency for improved GRFS, and had no influence on the relapse rate.

## 4. Venetoclax-Enhanced Sequential FLAMSA-RIC

Since the first evidence of venetoclax’s efficacy in an AML context was provided, several German transplant centers have started to add venetoclax to FLAMSA-RIC conditioning. This further led to a retrospective trial including 61 patients [41] (median age 58 years) treated for myeloid malignancies (40 AML, 18 MDS, 2 CMML, 1 CML) between 2018 and 2022. Amongst them, 74% had high-risk features. A total of 60 patients had active disease at the time of transplant (19 untreated, 41 relapsed/refractory), while three patients had been treated with previous transplant. SqC regimen consisted of the original FLAMSA-RIC protocol, FLAMSA-RIC (Treo), or FLAMSA-RIC (Mel). Total doses of venetoclax were highly variable since some patients were already using this medication. For others, venetoclax was added one to two days before FLAMSA and was pursued for seven to nine days. At day + 30, 91% of the patients achieved complete hematological remission (CR or CRi) and no delay in neutrophil engraftment was observed. At a median follow-up of 548 days, 44 (72%) of the patients were alive and 38 patients were in ongoing remission. Relapse occurred less frequently and later in the course of the disease for patients who were previously untreated compared to pretreated patients. With a 1-year TRM of 7%, FLAMSA-RIC + venetoclax is safe and feasible, and seems to be a good option for patients with high-risk or active myeloid malignancies. The relapse rate was still high (38% at day 172) but seems lower than that of patients treated with FLAMSA-RIC in the setting of R/R AML (52–69%) [13,41].

Studies with the addition of venetoclax to the conditioning regimen are ongoing (NCT05005299). The MD Anderson Cancer Center is currently conducting a randomized phase II trial studying the combination of venetoclax and sequential busulfan, cladribine, and fludarabine in patients with AML and MDS (NCT02250937).

## 5. Myelodysplastic Syndrome with Increased Blasts

Currently, allo-HSCT remains the only chance of cure for patients with MDS [42]. The optimal management of high-risk myelodysplastic syndrome has not yet been defined. Some retrospective studies have stated that the use of hypomethylating agents (HMAs) before allo-HSCT did not improve outcomes in comparison to upfront stem cell transplantation [43,44,45]. MDS often occurs in the elderly population and allo-HSCT carries a risk of toxicities and treatment-related mortality. More than the treatment itself, disease cytogenetics, primary refractory disease, and patient performance status seemed to be more relevant to the outcomes. In retrospective studies, RIC protocols showed decreased NRM compared to MAC; however, this was balanced by a higher risk of relapse [46,47] and lower OS [48]. A low disease burden, with bone marrow blasts < 5%, is associated with a reduced incidence of relapse after transplantation [49], but prospective studies are lacking and disease burden has not been proven to be a modifiable risk factor for MDS. Is SqC a good strategy to achieve a lower disease burden before transplantation without postponing it?

In 2023, a retrospective EBMT analysis compared SqC to MAC and RIC for patients with MDS with an excess of blasts at the time of allo-HSCT [50]. The sequential conditioning regimens used were FLAMSA or clofarabine-based. No significant differences were observed between the three strategies. The 3-year OS of the entire cohort was 50%, cumulative incidence of relapse was 27%, and NRM was 28%. Even though the probability to be alive at three years without previous GvHD or progression/relapse was higher for SqC compared to RIC and MAC (34%, 19%, and 18%, respectively), the multivariate analysis failed to demonstrate superiority concerning OS, relapse-free survival (RFS), and NRM. Relapse was borderline significantly higher in patients receiving SqC compared to MAC (HR 2.37; (95% CI; 1.03–5.40)). Once again, patient characteristics and disease cytogenetics seemed to be more relevant to post-transplant outcomes, and pre-transplant treatment did not seem to affect outcomes.

Another retrospective study on behalf of the SFGM-TC showed comparable OS and PFS when comparing FLAMSA-RIC (6.4 mg/kg Bu or 4 Gy Cy/TBI) to RIC (FluBu2) and MAC (FluBu3 or FluBu4) [51] in 427 patients allografted for high-risk MDS. In this analysis, patients with progressive disease or with excess marrow blasts (>10%) were more likely to be treated with SqC conditioning, and some of them did not receive ATG. Multivariate analysis showed a similar incidence of aGvHD stage II–IV between the three groups, but a higher incidence of extensive cGvHD in patients in the FLAMSA-RIC group, who also received more DLIs. The entire cohort’s 3-year OS and PFS were 50.4% and 43%, respectively. Again, the main factor influencing outcomes were the disease characteristics at diagnosis and not the treatment modality.

Thus, in the MDS setting, where outcomes are determined by patient and disease characteristics and not by the conditioning strategy, no advantage of the SqC strategy could be demonstrated. However, there may be some advantage, as one study [50] showed a lower incidence of GvHD with FLAMSA-RIC, which may allow more patients to be treated with pDLI. Another study [51] observed comparable OS and PFS for SqC, despite a trend towards a higher percentage of bone marrow blasts in this group. Thus, we believe that SqC should be evaluated with prospective analysis, with a focus on the feasibility of pDLI for patients with high-risk MDS eligible for allo-HSCT.

Newer sequential strategies are being studied. Among them, CPX-351 used as debulking chemotherapy followed by RIC after 5 days of rest [52] seems interesting in terms of the tolerability and efficacy of disease control. Thus, CPX-351 could be a good option to reduce treatment toxicity and further investigations should evaluate this approach for patients with high-risk MDS/MPN.

## 6. Sequential Conditioning and HLA-Haploidentical/Mismatched Unrelated Donors and the Use of PTCy

In 2015, Zoellner et al. [27] (Table 2) introduced SqC followed by HLA-haploidentical HSCT for patients with non-Hodgkin’s lymphoma (NHL) who had not achieved remission prior to transplantation. The regimen used was clofarabine followed by RIC with fludarabine and cyclophosphamide plus melphalan. The graft was unmanipulated and T-cell replete and GvHD prophylaxis consisted of post-transplant cyclophosphamide, tacrolimus, and mycophenolate mofetil. This small cohort of 16 heavily pretreated patients showed a good early response rate (94% at day + 30) and 1- and 2-year PFS of 56% and 50%, respectively. This approach was well tolerated, with a 1-year NRM of 19% and a low incidence of acute and chronic GvHD. Donor lymphocyte infusion was feasible in two of the relapsed patients and induced remission (one PR and one CR).

Since then, other sequential conditioning regimens associated with HLA-haploidentical HSCT have been applied to myeloid diseases, such as the aforementioned TEC-RIC [26]. Another cohort showed good results for SqC with FLAMSA or clofarabine for high-risk and R/R AML and MDS patients undergoing HLA-haploidentical stem cell transplant (SCT) [29] (Table 2). PTCy was used for GvHD prophylaxis and pDLI was used after immunosuppression withdrawal and in the absence of GvHD. This cohort had a longer follow-up and showed low NRM and GvHD incidence as well as relatively good outcomes in terms of survival and disease control. However, almost all patients without evidence of aGvHD relapsed.

In 2019, Doppelhammer et al. [30] retrospectively compared the outcome of sequential conditioning amongst 68 patients with high-risk AML (defined as primary refractory AML, secondary AML, or AML harboring genetic aberrations classified as intermediate–high or adverse according to the European Leukemia Network (ELN) risk classification [53]. In this series, 34 patients receiving haplo-SCT were pair-matched with patients receiving HLA-matched SCT. The sequential conditioning used was FLAMSA-RIC for HLA-matched SCT and clofarabine-RIC or FLAMSA-RIC for HLA-haploidentical-SCT. At 3-year follow-up, no statistical difference was observed between donor types with regard to the various allo-HSCT outcomes, except for a higher cumulative incidence of acute GvHD (≥grade II) in the HLA-matched group compared to the HLA-haploidentical group (50 ± 8% vs. 13 ± 4%; *p* < 0.001). The cytoreductive regimen (clofarabine vs. FLAMSA) did not have an impact on any of the outcomes.

Haplo-HSCT with FLAMSA-Bu/Mel SqC was retrospectively evaluated in 2021 [31] (Table 2). This conditioning was compared in the setting of HLA-matched and HLA-haploidentical SCT. In the Haplo-group, the incidence of NRM was higher (58%, *p* = 0.0003), with a higher incidence of hemorrhagic and infectious complications (*p* = 0.002). The strong disparity of NRM influencing the OS for the haploidentical group was not explained and not observed in other retrospective studies.

There are many differences between these evaluations. Therefore, comparisons are not easy. We consider it safe to conclude that sequential conditioning followed by haploidentical stem cell transplantation is safe, feasible, and probably as effective as standard MAC. Secondary to the use of ATG from the FLAMSA-RIC protocol in combination with PTCy, some studies have observed a lower incidence of aGvHD in patients treated with haploidentical HSCT [26,54]. Thus, haploidentical HSCT can provide rapid access to SCT and prompt treatment without the need to find a matched unrelated donor.

## 7. Cellular Therapies, Future Treatment Options, and Sequential Conditioning

T-cell-based immunotherapies using chimeric antigen receptor (CAR) T-cells or bispecific T-cell-engaging antibodies are potential options for patients with hematological malignancies. However, the development of immunotherapies for AML and MDS has been slow, mainly because of difficulties in finding safe and suitable targets. Several clinical trials are ongoing concerning bispecifics and CAR T-cell therapies with the following targets: CD33, CD123, CLL-1, FLT3, CD7, and CD70. Bispecific T-cell-engaging antibodies targeting CD33 appear to be of limited efficacy in AML [55], but association with a STING agonist may improve their efficacy [56].

The combination of CAR T-cell therapy and allo-HSCT has been evaluated in several trials for B-ALL, mostly using allo-HSCT as a consolidation treatment after CAR T-cell therapy [57,58]. In adult B-ALL, CAR T-cell therapy offers a good CR rate, but it seems that a better chance of cure is provided when CAR T-cell therapy is consolidated with subsequent allo-HSCT once patients have achieved remission [59].

In AML, the use of CAR T-cell targeting CLL-1 and CD7 appears to be safe and effective [60,61]. A small cohort of 10 heavily pretreated patients with CD7+ R/R AML showed manageable toxicities [61]. Of these patients, seven were previously treated with allo-HSCT and three of these underwent a second allo-HSCT after achieving CR.

Another small but interesting study used a form of SqC with haploidentical CD7 CAR T-cell followed by haploidentical HSCT after CAR T-cell therapy resulted in CRi [62]. All 10 patients were heavily pretreated and had active disease at the start of treatment (7 AML, 3 T-cell acute lymphoblastic leukemia/lymphoma). No additional pharmacological myeloablation or GvHD prophylaxis was added to the regimen prior to allo-HSCT, allowing the CAR T-cells to survive alongside the allogeneic stem cells. It is suggested that the long-lasting CD7 CAR T-cells could contribute to GvHD prophylaxis without compromising the GvL effect. Intergrating T-cell-based immunotherapies with allo-HSCT remains an experimental approach.

Other options for better disease control before conditioning regimens for allo-HSCT are under evaluation. Amongst them, the use of ^131^I-apamistamab before RIC (Flu/TBI) [63] seems to be an interesting strategy, and further analysis with well-conducted prospective randomized trials would allow an easier evaluation of its efficacy. Gemtuzumab-ozogamicin, the anti-CD33 conjugated antibody, has been associated with an increased risk of sinusoidal obstruction syndrome (SOS) [64]. However, after dose adaptations, more recent reports have documented the use of gemtuzumab-ozogamicin, even as part of a SqC, without an increased risk of SOS [65,66,67].

## 8. Discussion

Nearly all transplant physicians think that blast clearance before transplantation is of major importance. Other strategies aimed at reducing the burden of disease prior to conditioning are under investigation. For example, “preconditioning intervention” is an approach similar to SqC. A course of chemotherapy is administered and a reduced-intensity conditioning regimen is started within 14 days of chemotherapy withdrawal. Tachibana et al. (2025) [68] evaluated a small cohort of R/R AML patients treated with preconditioning intervention and showed very good results: a 2-year OS of 67.1%, 2-year CIR of 23.9%, and a 2-year NRM of 8.8%. However, the patient sample was small, the treatment approach was heterogeneous, and multivariate analysis could not be performed.

Interestingly, a recent study on patients with R/R AML analyzed the impact of early blast clearance after intensive chemotherapy with high-dose melphalan and before the administration of RIC [69]. Though achieving early blast clearance led to a significantly reduced NRM, it did not affect survival after allo-HSCT. It seems that effective blast reduction could not alleviate the increased risk of relapse in patients with a high leukemic burden before SqC and reverse their poor prognosis.

So far, no trial has shown a clear benefit of SqC. As observed when comparing Table 1 and Table 2. some outcomes may appear to be better after SqC, but randomized clinical trials are still lacking. At this time, we would not recommend SqC for high-risk MDS. For the same reason, MAC would be suggested for fit patients with R/R AML. However, in the case of older unfit patients with active disease, not eligible for MAC, SqC could be a feasible option to increase short-term disease control. Amongst the different SqC options, it seems that toxicity can be lowered with the use of TEC-RIC or FLAMSA-Treo, avoiding TBI.

We believe that in order to lower the relapse risk after allo-HSCT, every effort should be made to decrease toxicity, morbidity, and mainly GvHD. This will enable the physicians to use the different early relapse-preventing modalities in the post-transplant setting. In case of active disease before transplantation, patients in remission after allo-HSCT should be closely monitored and offered targeted maintenance therapy if available. Moreover, prompt immunosuppression tapering and prophylactic/preemptive treatment with donor lymphocyte infusions should be considered for all patients, regardless of the conditioning regimen used for allo-HSCT. The FIGARO trial [21] showed that the acquisition of full donor T-cell chimerism at three months after transplantation was associated with a lower cumulative incidence of relapse (13.1% vs. 44.8%) and with a comparable outcome, regardless of pretransplant MRD status. This could be achieved with better control of the disease before transplantation but also with the administration of pDLI. Thus, sequential conditioning in with pDLI seems to remain a good option for older patients or those with comorbidities.

A better overall survival after allo-HSCT is observed when allo-HSCT is performed earlier in the course of the disease. In addition, the administration of multiple lines of chemotherapy prior to allo-HSCT is associated with increased rates of aGvHD and decreased GvHD-relapse-free survival [70,71], negatively impacting post-transplant disease management and the use of (p)DLI.

Whether SqC is really associated with less GvHD has not been demonstrated. The lower GvHD incidence observed is probably related to ATG use. However, to improve post-transplant management and increase the use of pDLI, we encourage the use of ATG in the RIC part of SqC, even in combination with PTCy. The question of a loss of the GvL effect due to the use of ATG is often raised but, until now, the evidence has pointed towards the conclusion that ATG does not impact the graft-versus-leukemia effect [72,73,74]. DLI has been proven to improve chimerism and OS in patients with mixed chimerism after transplantation [75,76,77], but the studies mentioned in this article report a low incidence of pDLI use (0–25%) [18,24,26,27,30,31], mainly due to contraindications limiting the use of this strategy. Therefore, relying on pDLI would not be sufficient to improve the prognosis of patients with high-risk or R/R disease.

The use of DLI as a prophylactic or preemptive approach is associated with toxicities such as GvHD [75,76,77]. A better understanding of HLA evolutionary divergence (HED) and the use of the HED score may help to define which patients may benefit most from treatment with DLI and which patients are at a higher risk of toxicities [78]. The HED score seems to be an interesting tool to predict outcomes, as a high class II HED score seems to have a positive impact on OS and GRFS, and HED could help to identify patients at a higher risk of severe GvHD.

In view of the many unanswered questions regarding the management of R/R hematological malignancies prior to allo-HSCT, we believe that more prospective randomized trials should be conducted, not only on SqC and other pre-conditioning strategies to achieve remission, but also on the integration of different therapeutic options, with a particular focus on the feasibility of post-transplant therapies.

## Figures and Tables

**Table 1:** Outcomes of conditioning regimens for patients with active disease.

Authors	Disease—Age	Disease Status	Conditioning Regimen	OS	CIR	NRM
**Patients with Active Disease**						
Duval et al. (2010) [4]	ALL, AML Age 1–70 y	AD	TBI/Cyclophosphamide Busulfan/Cyclophosphamide	ALL: 16% AML: 19% (3 years)		
Nagler et al. (2015) [5]	AML Age 18–68 y	AD	Busulfan/Cyclophosphamide	31.2% (2 years)	53.5% (2 years)	21.5% (2 years)
			TBI/Cyclophosphamide	33.4% (2 years)	54% (2 years)	17.5% (2 years)
Henderson et al. (2021) [10]	AML, MDS, MPN, CML Age 18–69 y	AD: 55%	Treosulfan—Cytarabine/Fludarabine	46.2% (2 years)	37.6% (3 years)	20.9% (2 years)
Connor et al. (2023) [11]	AML, MDS, CML (AP), BPDCN Age 22–70 y	AD	Clofarabine/Busulfan (MAC)	40% (2 years)	39% (2 years)	31% (2 years)
Shimomura et al. (2024) [9]	AML Age 55–65 y	AD	Busulfan/Fludarabine (RIC) Melphalan/Fludarabine (RIC)	22% (5 years)	53.36% (5 years)	27.5% (5 years)

Abbreviations: OS, overall survival; CIR, cumulative incidence of relapse; NRM, non-relapse mortality; AML, acute myeloid leukemia; MDS, myelodysplastic neoplasm; MAC, myeloablative conditioning; RIC, reduced-intensity conditioning; TBI, total body irradiation; ALL, acute lymphoblastic leukemia; AD, active disease; MPN, myeloproliferative neoplasm; CML, chronic myelogenous leukemia; active phase; BPDCN, blastic plasmacytoid dendritic cell neoplasm.
